# The Ethiopian Third National Tuberculosis Drug Resistance Survey Incorporating Whole Genome Sequencing

**DOI:** 10.1093/ofid/ofaf367

**Published:** 2025-07-21

**Authors:** Shewki Moga, Muluwork Getahun, Zemedu Mohammed, Ayinalem Alemu, Getu Diriba, Bazezew Yenew, Dinka Fikadu, Yeshiwork Abebaw, Misikir Amare, Ephrem Tesfaye, Abebaw Kebede, Zelalem Yaregal, Abyot Meaza, Hilina Mollalign, Biniyam Dagne, Mengistu Tadesse, Waganeh Sinshaw, Getachew Seid, Betselot Zerihun, Melak Getu, Gemechu Tadesse, Saro Abdella, Getachew Tollera, Abayneh Admas, Addisalem Yilma, Yohannes Molla, Fekadesselassie Mikru, Dawit Assefa, Tefera Girma, Beniam Feleke, Federico Di Marco, Daniela M Cirillo, Anna Dean, Andrea Maurizio Cabibbe, Eveline Klinkenberg

**Affiliations:** Infectious Diseases Research Directorate, Ethiopian Public Health Institute, Addis Ababa, Ethiopia; Infectious Diseases Research Directorate, Ethiopian Public Health Institute, Addis Ababa, Ethiopia; Infectious Diseases Research Directorate, Ethiopian Public Health Institute, Addis Ababa, Ethiopia; Infectious Diseases Research Directorate, Ethiopian Public Health Institute, Addis Ababa, Ethiopia; Infectious Diseases Research Directorate, Ethiopian Public Health Institute, Addis Ababa, Ethiopia; Infectious Diseases Research Directorate, Ethiopian Public Health Institute, Addis Ababa, Ethiopia; Infectious Diseases Research Directorate, Ethiopian Public Health Institute, Addis Ababa, Ethiopia; Infectious Diseases Research Directorate, Ethiopian Public Health Institute, Addis Ababa, Ethiopia; Infectious Diseases Research Directorate, Ethiopian Public Health Institute, Addis Ababa, Ethiopia; Infectious Diseases Research Directorate, Ethiopian Public Health Institute, Addis Ababa, Ethiopia; Infectious Diseases Research Directorate, Ethiopian Public Health Institute, Addis Ababa, Ethiopia; Infectious Diseases Research Directorate, Ethiopian Public Health Institute, Addis Ababa, Ethiopia; Infectious Diseases Research Directorate, Ethiopian Public Health Institute, Addis Ababa, Ethiopia; Infectious Diseases Research Directorate, Ethiopian Public Health Institute, Addis Ababa, Ethiopia; Infectious Diseases Research Directorate, Ethiopian Public Health Institute, Addis Ababa, Ethiopia; Infectious Diseases Research Directorate, Ethiopian Public Health Institute, Addis Ababa, Ethiopia; Infectious Diseases Research Directorate, Ethiopian Public Health Institute, Addis Ababa, Ethiopia; Infectious Diseases Research Directorate, Ethiopian Public Health Institute, Addis Ababa, Ethiopia; Infectious Diseases Research Directorate, Ethiopian Public Health Institute, Addis Ababa, Ethiopia; Infectious Diseases Research Directorate, Ethiopian Public Health Institute, Addis Ababa, Ethiopia; Infectious Diseases Research Directorate, Ethiopian Public Health Institute, Addis Ababa, Ethiopia; Health Laboratory Services, Ethiopian Public Health Institute, Addis Ababa, Ethiopia; Public Health Research, Ethiopian Public Health Institute, Addis Ababa, Ethiopia; National TB and Leprosy Control Program, Ministry of Health, Addis Ababa, Ethiopia; Country Office for Ethiopia, World Health Organization, Addis Ababa, Ethiopia; Challenge TB Project, United States Agency for International Development, Addis Ababa, Ethiopia; Country Office for Ethiopia, World Health Organization, Addis Ababa, Ethiopia; Challenge TB Project, United States Agency for International Development, Addis Ababa, Ethiopia; Division of Global HIV and Tuberculosis, US Centers for Disease Control and Prevention, Addis Ababa, Ethiopia; Division of Global HIV and Tuberculosis, US Centers for Disease Control and Prevention, Addis Ababa, Ethiopia; Division of Immunology, Transplantation, and Infectious Diseases, IRCCS San Raffaele Scientific Institute, Milan, Italy; Division of Immunology, Transplantation, and Infectious Diseases, IRCCS San Raffaele Scientific Institute, Milan, Italy; Global TB Program, World Health Organization, Geneva, Switzerland; Division of Immunology, Transplantation, and Infectious Diseases, IRCCS San Raffaele Scientific Institute, Milan, Italy; Challenge TB Project, United States Agency for International Development, Addis Ababa, Ethiopia; Global TB Program, World Health Organization, Geneva, Switzerland; Department of Global Health, Amsterdam University Medical Centers, Amsterdam, The Netherlands

**Keywords:** drug resistance survey, drug-resistant TB, Ethiopia, tuberculosis, whole genome sequencing

## Abstract

**Background:**

Drug-resistant tuberculosis (DR-TB) is a major challenge hindering global tuberculosis control. Ethiopia conducted a third national antituberculosis (TB) drug resistance survey, and this is the first survey to report on drug resistance using whole genome sequencing (WGS) in addition to genotypic and phenotypic test results. The aim of this study was to obtain up-to-date information regarding the magnitude and pattern of drug resistance in Ethiopia.

**Methods:**

A nationwide cross-sectional study was conducted in 217 health facilities across all Ethiopian regional states from August 2017 to January 2019. Sputum specimens were collected from patients with bacteriologically confirmed pulmonary TB to detect resistance to anti-TB drugs with Xpert MTB/RIF assay, culture-based phenotypic drug susceptibility testing (DST), and WGS with phylogenetic analysis.

**Results:**

The prevalence of rifampicin-resistant TB (RR-TB) was 1.07% (95% confidence interval [CI], .65%–1.74%) among new cases and 6.89% (95% CI, 4.02%–11.57%) among previously treated cases. The prevalence of isoniazid-resistant, rifampicin-susceptible TB was 4.15% (95% CI, 3.11%–5.53%) among new cases and 4.41% (95% CI, 1.97%–9.57%) among previously treated cases. While resistance to fluoroquinolones was detected in 1 RR-TB case, resistance to bedaquiline and linezolid was not detected in RR-TB cases. *Mycobacterium tuberculosis* lineage 4 was the most common, followed by lineage 3 and lineage 1, with sublineage 4.2.2 being the most frequent.

**Conclusions:**

The level of RR-TB remained low. Expanding baseline DST for isoniazid may help further lower the burden of DR-TB in Ethiopia.

Tuberculosis (TB) is a communicable disease that remains a major public health concern globally. According to the World Health Organization's (WHO) Global TB Report 2024, an estimated 10.8 million people developed TB and 1.25 million died from the disease in 2023 worldwide [1]. The occurrence of drug-resistant TB (DR-TB) has further complicated TB control measures. In 2023, there were an estimated 400 000 cases of rifampicin (RIF)–resistant TB (RR-TB) and multidrug-resistant TB (MDR-TB), accounting for 3.7% of all TB cases globally. In the same year, 15.4% of notified RR-/MDR-TB cases were extensively drug-resistant TB (XDR-TB) or pre-XDR-TB [1]. In 2021, an estimated 1.1 million (11% [range, 6.5%–15%]) people with TB had isoniazid (INH)–resistant, RIF-susceptible TB (Hr-TB) globally, much higher than the estimated burden of RR-/MDR-TB [2].

Ethiopia is among the 30 countries with a high TB and TB/human immunodeficiency virus (HIV) burden, with an estimated TB incidence of 146 (95% confidence interval [CI], 98–203) per 100 000 population in 2023 [3]. Molecular WHO-recommended rapid diagnostics (mWRDs) are being used for the diagnosis of presumptive TB and DR-TB cases in Ethiopia [4]. However, the diagnosis of TB still relies heavily on smear microscopy and clinical decision, and drug susceptibility testing (DST) is not yet universally available for screening all TB patients for drug resistance. In 2022, only 32% of people notified with TB had received an mWRDs as the initial diagnostic test [5]. In the same year, 74% of new bacteriologically confirmed cases were tested for RIF resistance and 54% of people notified with RR-TB were tested for fluoroquinolone (FLQ) resistance [5]. Due to gaps in routine testing, the burden and pattern of drug-resistant TB in resource-limited settings such as Ethiopia are commonly determined through periodic drug resistance surveys (DRSs) conducted every 5 years [6].

A DRS is critical for measuring the progress of the national program in controlling DR-TB and identifying programmatic intervention areas. Ethiopia conducted its first nationwide DRS from 2003 to 2005, indicating MDR-TB prevalence of 1.6% (95% CI, .9%–2.8%) among new and 11.8% (95% CI, 5.6%–21%) among previously treated TB patients [7]. Between 2011 and 2013, the country conducted the second round of DRS indicating MDR-TB prevalence of 2.3% (95% CI, 1.5%–3.1%) and 17.8% (95% CI, 13.2%–22.4%) among new and previously treated cases, respectively [8]. However, the design of the second DRS was not considered nationally representative since it was conducted in the same sites as the first DRS despite major changes to the coverage of the health system, with the establishment of many additional health facilities. Therefore, a third DRS was conducted in order to obtain updated and nationally representative data on prevalence of DR-TB in Ethiopia. Additionally, this is the first survey in the country to provide a deeper understanding of the DR-TB patterns and resistance-conferring mutations using whole genome sequencing (WGS).

## MATERIALS AND METHODS

### Study Design, Sample Size, and Sampling Method

A health facility–based, cross-sectional survey was conducted from August 2017 to January 2019 at randomly selected health facilities across all regions in Ethiopia. The study population included bacteriologically confirmed new and previously treated pulmonary TB (PTB) cases of all age groups diagnosed at survey sites using either Ziehl-Neelsen smear microscopy or Xpert MTB/RIF assay. The sample size was estimated based on single proportion estimation using cluster sampling strategy according to the WHO guidance [6]. Based on an assumed RR-TB prevalence of 2.5%, a required absolute precision of 1% at the 95% CI, a cluster sampling design effect of 2, and an expected loss of 10%, a total of 2010 bacteriologically confirmed new PTB cases were calculated as the target sample size.

A cluster sampling strategy was used to select survey sites from the total of 4503 TB diagnostic health facilities in the country in 2016. At the time of the survey, the Ethiopian government structure was composed of 9 regional states (Afar, Amhara, Benishangul-Gumuz, Gambella, Harari, Oromia, Somali, Southern Nation Nationalities and Peoples, and Tigray) and 2 city administrations (Addis Ababa and Dire Dawa). The regional states and city administrations are further divided into *woredas* (districts) and *kebeles* (subdistricts). Most regions have zones above the woredas level as subregional administrative structures of the regional states. A health facility or group of health facilities with annual smear-positive PTB case notification of ≥30 constituted a cluster. From a total of 1237 clusters included in sampling frame, 67 clusters comprising 217 diagnostic health facilities were randomly selected using probability proportional to size sampling.

### Participant Recruitment and Data Collection

In this DRS, TB cases were identified at TB clinics in selected health facilities based on the standard diagnostic approaches outlined in the national guidelines [9]. Consecutive PTB cases newly registered for treatment were invited to participate in the survey based on written informed consent/assent, and those who provided consent were enrolled in the survey. All previously treated cases registered for treatment during the study period were also included in the survey. Patients who had received their ongoing course of treatment for >1 week at the time of enrollment were excluded, as were patients with extrapulmonary TB and clinically diagnosed patients. Demographic, clinical, behavioral, and socioeconomic data were collected at enrollment for each study participant through interview and review of medical records.

### Drug Susceptibility Testing

For each eligible TB patient, 1 morning sputum specimen (2–5 mL) was collected upon enrollment and transported to the regional TB culture laboratories (RTCLs) for culture and Xpert MTB/RIF assay, while phenotypic DST (pDST) was done at the National TB Reference Laboratory (NTRL) of the Ethiopian Public Health Institute (EPHI) located in Addis Ababa. For cases enrolled from regions where TB culture laboratories were not available or not linked to RTCLs, the collected sputum sample was directly transported to the NTRL for Xpert MTB/RIF assay, culture, and pDST.

For culture isolation of *Mycobacterium tuberculosis* complex (MTBC), specimens were liquefied and decontaminated as previously described [10]. The pellet was then inoculated into both Löwenstein-Jensen medium and the Mycobacteria Growth Indicator Tube 960 (MGIT 960) system (Becton Dickinson, Sparks, Maryland, USA). The Xpert MTB/RIF assay was also performed on the pellet of processed sputum sediment using a sample-to-reagent ratio of 1:3 as previously described [11].

Phenotypic DST for first-line drugs was done for all participants with pure culture isolates using the MGIT 960 system according to the WHO-recommended critical concentrations of 0.1 µg/mL for INH, 1.0 µg/mL for RIF, 5 µg/mL for ethambutol (EMB), and 100 µg/mL for pyrazinamide (PZA) [12]. The detailed pDST procedures were performed as previously described [10]. GenoType MTBDR*plus* VER 2.0 line probe assay (LPA) was conducted on participants who did not have pDST or WGS results. All quality assurance measures were considered for each testing procedure. The NTRL participates in annual proficiency testing scheme provided by the Ugandan WHO TB Supranational Reference Laboratory (SRL), formerly by the SRL in Milan (Italy), with documented evidence of acceptable performance in DST for all test methods and drugs throughout its participation.

### Whole Genome Sequencing

**Figure 1.**
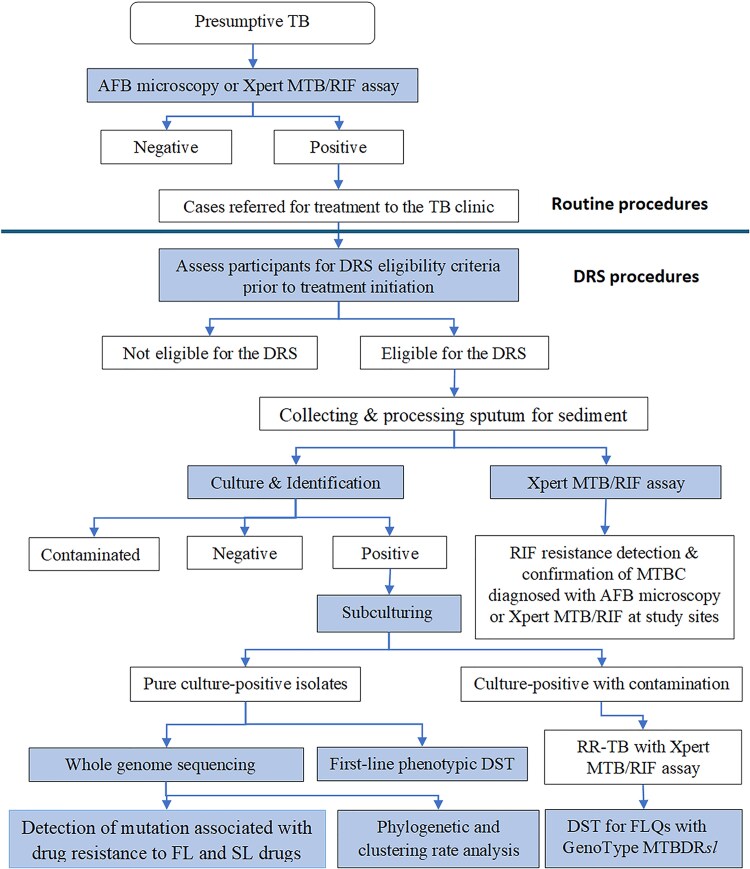
Flowchart of patient selection and test methods used in drug resistance surveys in Ethiopia, 2017–2019. Abbreviations: AFB, acid-fast bacilli; DRS, drug resistance survey; DST, drug susceptibility testing; FL, first-line; FLQs, fluoroquinolones; MTBC, *Mycobacterium tuberculosis* complex; RIF, rifampicin; RR-TB, rifampicin-resistant tuberculosis; SL, second-line; TB, tuberculosis.

WGS was performed on samples from cultures to detect resistance-conferring mutations for all identified RR-TB and Hr-TB isolates. In addition, a random sample of one-fourth of INH/RIF-susceptible MTBC strains was sequenced. The culture isolates were heat inactivated, at NTRL, and sent for sequencing to the WHO SRL in Milan. MTBC genomic DNA was extracted and purified using the Maxwell 16 Instrument (Promega), according to the manufacturer's instructions. Nextera XT DNA Library Preparation Kit and NextSeq/MiniSeq reagent kits (Illumina, San Diego, California, USA) were used as instructed by the manufacturer and libraries were sequenced on NextSeq 500 or MiniSeq platforms. The MTBseq pipeline was used to map sequence reads to the H37Rv genome (GenBank accession number NC_000962.3) [13] and calling variants at 10% minimum variant frequency [14]. WGS drug resistance results were analyzed and interpreted using the updated 2023 WHO catalogue of mutations [15]. The final RIF-DST result was interpreted based on the WGS result. For RR-TB cases where WGS was not available due to a lack of pure culture isolates, the RIF resistance result of Xpert MTB/RIF assay was considered the final result, and resistance to FLQ was determined by GenoType MTBDR*sl* LPA (Figure 1). Lineage and single-nucleotide polymorphism (SNP) differences were analyzed to visualize the genetic diversity and relatedness among MTBC isolates. An SNP cut-off of ≤5 SNPs was adopted to define genetic relatedness, implying likely recent transmission [16]. Recent transmission index (RTI) was calculated to infer clustering rate using the formula RTI = (nc – c) / n, where “n” is the total number of cases, “nc” is the number of cases within clusters (groups of 2 or more cases), and “c” is the total number of clusters. WGS data are available in the Sequence Read Archive of the National Center for Biotechnology Information as fastq files, under study accession number PRJNA1104194.

### Data Management and Statistical Analysis

Data were collected on paper at the enrolling health facilities. Preprinted barcodes representing unique individual identifiers were used to label each form and sample. Data entry of the patient forms was done at the central level at the data management unit of EPHI in a specially designed EpiInfo database. Barcodes were scanned to avoid transcription errors. Analysis was performed using SPSS statistics version 20 (IBM Corporation, Armonk, New York, USA) and Stata version 15.0 (StataCorp, College Station, Texas, USA) software. Descriptive analysis was performed to summarize the data, assess its distribution, identify patterns and trends, and analyze missing values. The combined results from all available DST methods (ie, Xpert MTB/RIF assay, pDST, LPA, or WGS) were used to define RIF resistance. In case of discordant findings, all results were triple-checked and reascertained. In case neither of these DST methods provided a valid result for RIF, the patients was classified as having unknown RIF outcome status. The standardized WHO approach for analyzing DRS surveys [17] was used for estimating RR-TB separately for both new and retreatment cases at the cluster and individual level using logistic regression with robust standard errors by taking clustering into account. To account for over/underenrollment, weighing was done relative to the target cluster size of 30. Imputation models were developed to account for those missing RIF outcomes. Following prevalence estimations for RIF resistance, the same approach was used for estimating the prevalence of INH resistance, including both any INH resistance and Hr-TB. Descriptive statistics were computed for new and previously treated cases. Binary logistic regression was computed to identify the potential risk factors for RR-TB. The odds ratio with 95% CI was used to measure the strength of association between RR-TB cases and predictor variables. A *P* value of <.05 in the adjusted model was considered statistically significant.

### Ethical Considerations

This study was reviewed and approved by the EPHI Institutional Review Board. Participants were informed about the study and asked to provide written informed consent before participation. Assent with parental/guardian consent were obtained for participants between 10 and 18 years of age.

## RESULTS

A total of 2560 (2267 new and 293 previously treated) patients with PTB were enrolled in this survey, of whom 2148 (83.9%) were included based on a positive smear microscopy and 412 (16.1%) participants based on a positive Xpert MTB/RIF assay. Of the 2560, 284 (11.1%) who were initially diagnosed using acid-fast bacilli microscopy at the health facility were excluded as *M tuberculosis* (MTB) was not confirmed upon retesting with Xpert MTB/RIF at RTCLs/NTRL (Figure 1). Therefore, 2276 TB cases, 2042 new and 234 previously treated, were included in the DRS analysis (Table 1).

**Table 1.** Characteristics of Participants in the Third National Drug Resistance Survey in Ethiopia, 2017–2019

Characteristic	Newly Diagnosed	Previously Treated	Total
	(n = 2036)	(n = 234)	(n = 2276)
Sex			
Male	1150 (56.3)	142 (60.7)	1292 (56.8)
Female	892 (43.7)	92 (39.3)	984 (43.2)
Age group, y			
<15	99 (4.8)	3 (1.3)	102 (4.5)
15–24	728 (35.7)	48 (20.5)	776 (34.1)
25–34	608 (29.8)	71 (30.3)	679 (29.8)
35–44	295 (14.5)	45 (19.2)	340 (14.9)
45–54	177 (8.7)	37 (15.8)	214 (9.4)
55–64	77 (3.8)	13 (5.6)	90 (4.0)
≥65	58 (2.8)	17 (7.3)	75 (3.3)
Residence			
Rural	1302 (63.8)	131 (56.0)	1433 (63.0)
Urban	740 (36.2)	103 (44.0)	843 (37.0)
HIV status			
Negative	1889 (92.5)	190 (81.2)	2079 (91.3)
Positive	136 (6.7)	41 (17.5)	177 (7.8)
Unknown	17 (0.8)	3 (1.3)	20 (0.9)
Known diabetes			
Yes	21 (1.0)	6 (2.6)	27 (1.2)
No	2021 (99.0)	228 (97.4)	2249 (98.8)
TB contact			
Yes	593 (29.1)	73 (31.2)	666 (29.3)
No	1449 (71.0)	161 (68.8)	1610 (70.7)
DR-TB contact			
Yes	25 (1.2)	6 (2.6)	31 (1.4)
No	2017 (98.8)	228 (97.4)	2245 (98.6)

Data are presented as No. (%).

Abbreviations: DR-TB, drug-resistant tuberculosis; HIV, human immunodeficiency virus; TB, tuberculosis.

Just over half of the participants (56.8%) were male and 43.2% were female. The majority (78.8%) of participants were aged 15–44 years, with a median age of 27 (interquartile range, 20–37) years. HIV status was available for almost all participants (99.1%), with 7.8% testing positive. A history of contact with known TB patient was mentioned by 29.3% of the study participants while 1.4% participants mentioned a history of contact with a known MDR-TB patient (Table 1). Supplementary Table 1 provides information on the sociobehavioral characteristics of the study participants.

### Drug Resistance Patterns of Study Participants to First-line Anti-TB Drugs

**Figure 2.**
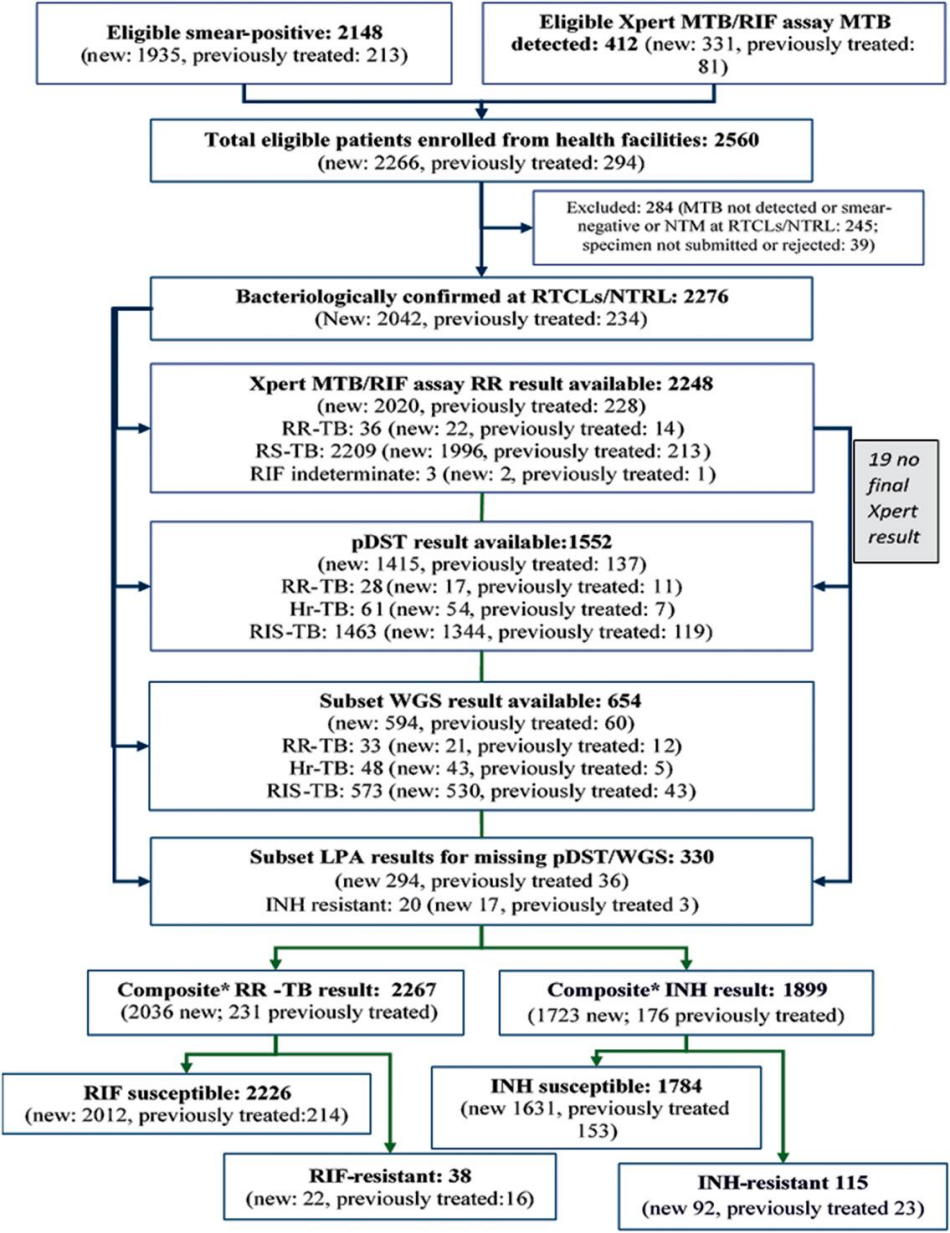
Flowchart of patient enrollment and drug resistance patterns included in the third national drug resistance survey in Ethiopia, 2017–2019. *Composite drug susceptibility testing (DST) consists of Xpert MTB/RIF assay, line probe assay, whole genome sequencing, and phenotypic DST result. Abbreviations: Hr-TB, isoniazid-resistant, rifampicin-susceptible tuberculosis; INH, isoniazid; LPA, line probe assay; MTB, *Mycobacterium tuberculosis*; NTM, nontuberculous mycobacteria; NTRL, national tuberculosis reference laboratory; pDST, phenotypic drug susceptibility testing; RIF, rifampicin; RIS-TB, rifampicin- and isoniazid-susceptible tuberculosis; RR, rifampicin resistance; RR-TB, rifampicin-resistant tuberculosis; RS-TB, rifampicin-susceptible tuberculosis; RTCLs, regional tuberculosis culture laboratories; WGS, whole genome sequencing.

Of 2276 TB cases who were bacteriologically confirmed upon retesting, DST results were available for 2267 (99.6%) cases (2036 new and 231 previously treated) for analysis of drug resistance patterns. Among bacteriologically confirmed cases, RIF resistance result was available for 2248 (98.8%) patients with Xpert MTB/RIF assay while pDST for INH, RIF, and EMB was available for 1552 (68.2%) patients and for PZA was available for 1482 (65.1%) patients. WGS results were available for 654 of 2276 (28.7%) patients, comprising patients with RR-/MDR-TB, Hr-TB, and non-RR-TB (Figure 2).

**Figure 3.**
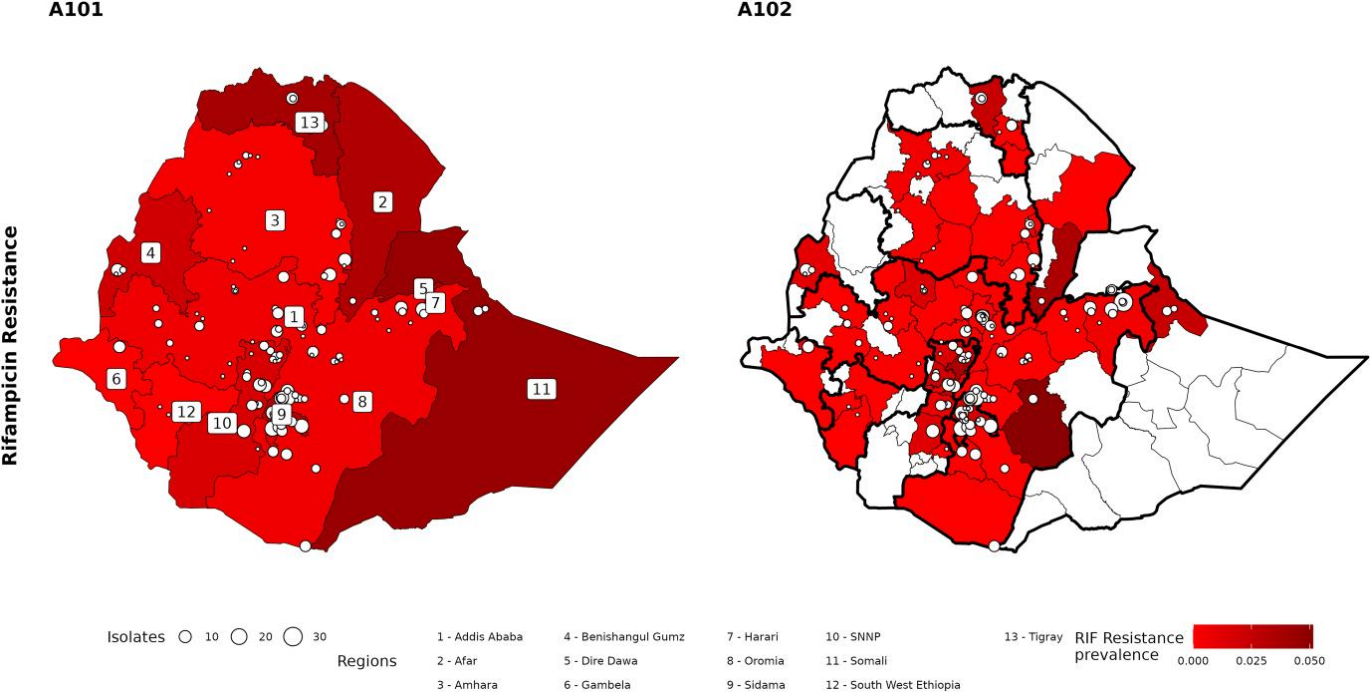
Geographic distribution of rifampicin-resistant tuberculosis cases (n = 38) among 2267 participants with rifampicin susceptibility testing results in the third national drug resistance survey in Ethiopia, 2017–2019. Abbreviations: A101, regional level; A102, zonal level; RIF, rifampicin; SNNP, Southern Nation Nationalities and Peoples.

Among 2267 patients with RIF susceptibility testing results, RR-TB was detected in 38 (1.7%) patients: 22 among new and 16 among previously treated patients. The prevalence of RR-TB was 1.07% (95% CI, .65%–1.74%) among new patients and 6.89% (95% CI, 4.02%–11.57%) among previously treated TB patients (Table 2). Supplementary Table 2 provides the model outcomes without imputation for MDR-TB and RR-TB prevalence. Of the 38 participants with RR-TB, WGS detected RIF resistance in 3 cases that were missed by Xpert MTB/RIF assay and in 2 cases that were missed by pDST. Supplementary Table 3 summarizes the discordance in RIF resistance among isolates tested with WGS, Xpert MTB/RIF assay, and pDST. Though the survey was not designed to produce subnational- or regional-level estimation, the proportion of TB patients with RIF resistance was highest in Addis Ababa (5.2%), Somali (4.6%), Tigray (3.9%), and Dire Dawa (3.9%) regions, acknowledging possible overestimation in certain regions where the survey recruitment rate was lower, for example, Somali and Dire Dawa (Figure 3, left).

**Table 2.** Prevalence of Resistance to First-line Antituberculosis Drugs by Treatment History Among Patients With Bacteriologically Confirmed Pulmonary Tuberculosis From the Third National Drug Resistance Survey in Ethiopia, 2017–2019

Resistance	Newly Diagnosed			Previously Treated		
	Total Tested, No.	Resistance, No. (%)	95% CI	Total Tested	Resistance, No. (%)	95% CI
RR-TB	2036	22 (1.07)	.65–1.74	231	16 (6.89)	4.02–11.57
MDR-TB	1723	21 (1.28)	.78–2.10	176	15 (8.4)	4.68–14.65
INH resistant	1723	92 (5.35)	4.14–6.88	176	23 (12.5)	8.10–18.88
Hr-TB	1723	71 (4.15)	3.11–5.53	176	8 (4.41)	1.97–9.57
EMB resistant	1428	35 (2.45)	1.53–3.23	140	10 (7.14)	3.26–11.02
PZA resistant	1361	29 (2.13)	1.01–2.92	135	4 (2.96)	.26–5.67

Abbreviations: CI, confidence interval; EMB, ethambutol; Hr-TB, isoniazid-resistant, rifampicin-susceptible tuberculosis; INH, isoniazid; MDR-TB, multidrug-resistant tuberculosis; PZA, pyrazinamide; RR-TB, rifampicin-resistant tuberculosis.

INH resistance was detected in 115 patients with TB (92 new and 23 previously treated) (Table 2). The estimated prevalence of any INH resistance was 5.35% (95% CI, 4.14%–6.88%) among new and 12.5% (95% CI, 8.10%–18.88%) among previously treated TB patients. Hr-TB was detected in 79 (71 new and 8 previously treated) TB patients (Table 2). The prevalence of Hr-TB was 4.15% (95% CI, 3.11%–5.53%) in new cases and 4.41% (95% CI, 1.97%–9.57%) in previously treated cases. Supplementary Table 4 provides the model outcomes without imputation for INH prevalence. Among the 115 INH-resistant isolates, 12 were identified only by pDST, whereas 2 were detected by WGS alone. Supplementary Table 5 summarizes the discordance in INH resistance between pDST and WGS.

### Drug Resistance Patterns of Study Participants to Second-line Anti-TB Drugs

Second-line DST results were available for 37 of 38 RR-TB cases; 33 of them had prediction for resistance mutation by WGS, and 4 by second-line LPA. Pre-XDR-TB was detected in 1 RR-TB case, a 25-year-old man with a history of previous TB treatment. XDR-TB was not detected in this survey. Clofazimine and bedaquiline resistance was detected in 1 patient with newly diagnosed non-RR-TB.

### Factors Associated With RR-TB

The prevalence of RR-TB resistance was significantly higher in patients previously treated with anti-TB drugs (odds ratio [OR], 6.38; *P* < .0001; Table 3). When looking at new cases only, contact with DR-TB (OR, 8.85 [95% CI, 1.91–41.08]; *P* = .005) and urban residence (OR, 2.95 [95% CI, 1.19–7.27]; *P* = .019) were significantly associated with having RR-TB.

**Table 3.** Risk Factors Associated With Rifampicin-Resistant Tuberculosis in the Third National Drug Resistance Survey in Ethiopia, 2017–2019

Risk Factor	Multivariate Logistic Regression Analysis	
	OR (95% CI)	*P* Value
Previous TB treatment history		
New	1	
Previously treated	6.38 (3.3–12.3)	**<**.**0001**
DR-TB contact		
No	1	
Yes	3.78 (.83–17.4)	.087
Formal schooling		
Yes	1	
No	1.79 (.94–4.10)	.073

A bold font, in the *P* value column, indicates a significant value (*P* < .05).

Abbreviations: CI, confidence interval; DR-TB, drug-resistant tuberculosis; MDR-TB, multidrug-resistant tuberculosis; OR, odds ratio; TB, tuberculosis.

### WGS-Based Phylogenetic Analysis and Clustering Rate of TB

**Figure 4.**
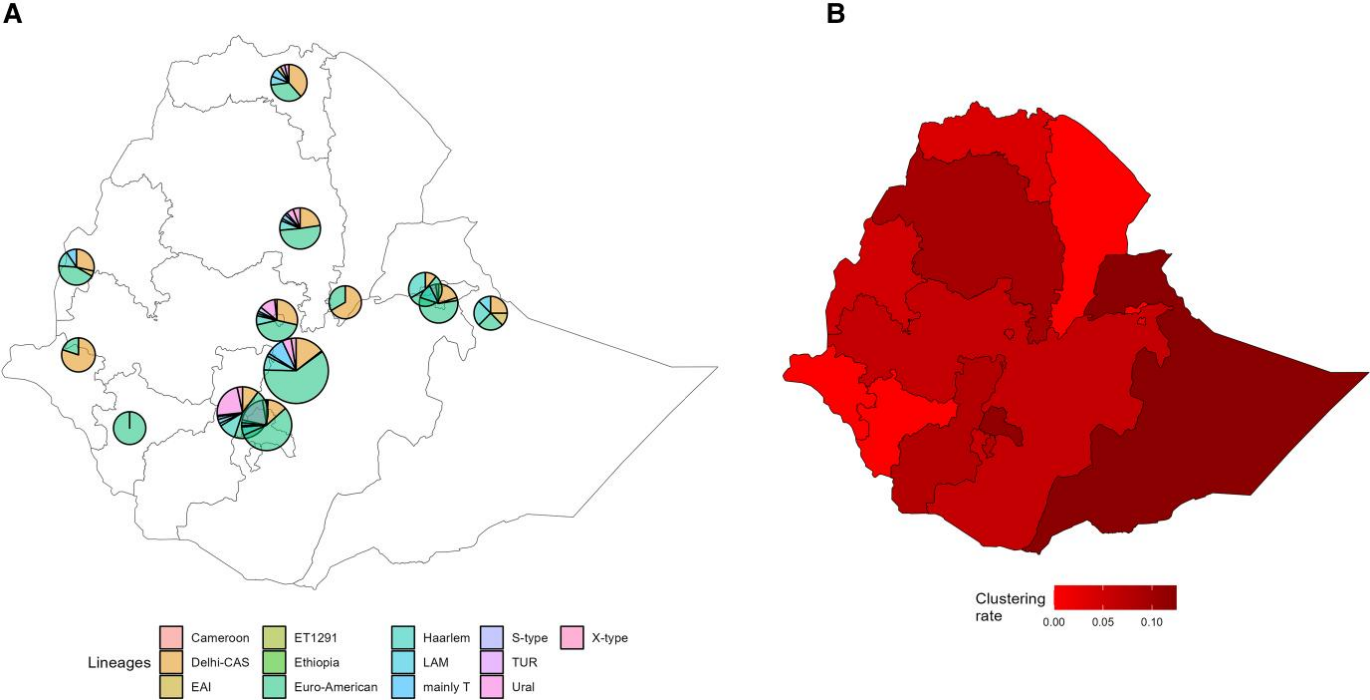
Whole genome sequencing–based phylogeographic distribution of 654 *Mycobacterium tuberculosis* (MTB) strains by region (A101) in Ethiopia during the third drug resistance survey, 2017–2019. *A*, MTB sublineage distribution (%). *B*, Clustering rate (%) at 5 single-nucleotide polymorphism distance threshold to define recent transmission chains.

MTB lineage 4 was the most common lineage nationwide (529/654 [80.9%]), followed by lineage 3 (120/654 [18.3%]) and lineage 1 (3/654 [0.5%]) (Figure 4*A*). Lineage 7 and the newly described ET1291 [18] were also detected once in Oromia and Tigray, respectively. Lineage 4 was the most prevalent in all regions except for Afar and Gambella, where lineage 3 was predominant. Within lineage 4, the Euro-American sublineage 4.2.2 was the most represented sublineage (232/654 [35.5%]).

The clustering rate (ie, RTI) of the strain collection subjected to WGS analysis at a 5 SNP distance threshold was 9.8%, highest in Somali (12.5%), Sidama (11.7%) and Amhara (9.4%) (Figure 4*B*). The clustering rate of the 33 cases with at least RIF resistance confirmed by WGS was 24.2%.

## DISCUSSION

The third DRS in Ethiopia demonstrated a low RR-TB prevalence of 1.1% among new patients and 6.9% among previously treated patients with TB. In the first DRS, a similarly low prevalence of RR-TB was observed among new cases at 1.6% [7], suggesting that the prevalence of RR-TB has remained stable over time. These findings are in line with the 2023 WHO estimate for MDR-/RR-TB in Ethiopia, which reported 1.1% among new cases [3], and are consistent with RR-TB prevalence reported in Kenya (1.3% new, 5.2% previously treated) and Eritrea (2.0% new, 7.6% previously treated) [19, 20], but considerably lower than the prevalence observed in Somalia (8.7% new, 43.2% previously treated) and Djibouti (4.7% new, 34.9% previously treated) [21, 22]. Additionally, the prevalence observed among previously treated cases in this survey was lower than the 11.8% reported in the first DRS [7]. The observed low level of RR-TB could be attributed to a high TB treatment success rate for DR-TB [23], community-based TB case finding, rapid expansion of mWRD-based DST services, adoption of a broader algorithm for detection of RR-TB in all bacteriologically confirmed TB cases [4], and improved access to second-line drug treatment services.

In recent years, there has been more global focus on the burden of Hr-TB, which has been shown to be more prevalent than RR-TB, and precedes RIF resistance in almost all MDR-TB cases [24]. In Ethiopia, Hr-TB was observed at 4.1% among new cases, which is about 3 times the RR-TB prevalence. This prevalence is comparable to that observed in Kenya (4.2%) and Eritrea (3.4%) [19, 20]. Hr-TB cases are more likely to result in amplification to MDR-TB compared to INH-susceptible TB cases [24]. However, unlike RR-TB, which can be diagnosed with MTB using Xpert MTB/RIF assay, the diagnosis of Hr-TB in the country is often delayed due to performing of DST for INH as a reflex test after ruling out RIF resistance and due to unavailability of rapid mWRDs capable of simultaneously detecting both INH and RIF resistance alongside MTB at the health facility level. Overall, our results indicate the need to implement rapid molecular diagnostics for the detection of INH resistance at baseline.

In this survey, we found only 1 participant with pre-XDR-TB in a person with previous history of treatment, amounting to 2.6% of the reported RR-TB cases. The strain belonged to the Euro-American 4.2.2 lineage with resistance to EMB and ethionamide, in addition to RIF, INH, and levofloxacin/moxifloxacin. In 2022, the country notified 8 pre-XDR-TB cases among 357 RR-/MDR-TB cases routinely tested for FLQ resistance corresponding to 2.2% of notified RR-/MDR-TB cases [5]. In the DRS conducted in neighboring Eritrea, 1 pre-XDR-TB case was reported among 14 patients with RR-TB and none were identified from the Djibouti survey [20, 22]. The lower prevalence observed in our survey could be due to judicious use of second-line drugs, effective and supervised treatment, and culture-based follow-up of RR-/MDR-TB cases in Ethiopia. In 2022, Ethiopia tested only 54% of RR-TB cases for FLQ resistance due to limited access to DST [5]. In 2023, the country has introduced a low-complexity mWRD, Xpert MTB/XDR test, for the rapid detection of FLQ resistance among all bacteriologically confirmed RR-TB cases at all RR-/MDR-TB treatment initiation centers to ensure access to baseline DST to FLQs prior to treatment.

This is the first survey to report on XDR-TB using the new WHO definition [1]. Ethiopia has implemented a fully oral short treatment regimen composed of bedaquiline, pretomanid, and linezolid with or without moxifloxacin (BPaL/BPaLM) for the treatment of RR-/MDR-TB cases since September 2023. In this survey, resistance to bedaquiline and linezolid was not detected in patients with RR-/MDR-TB. However, in 2024, the first case of acquired resistance to bedaquiline has been reported among RR-TB cases during treatment with the BPaLM regimen in Ethiopia [25]. Interestingly, we found resistance to bedaquiline and clofazimine in 1 non-RR-TB patient with WGS results (mutation Arg156* in Rv0678 gene at 29% frequency, X-type 4.1.1.3 strain). These findings highlight the need to monitor the spread of drug resistance to new second-line drugs, particularly bedaquiline and linezolid.

This survey represents the largest genomic collection of drug-susceptible and drug-resistant strains from the country and also marks the first time WGS results have been included in the DRS. In this regard, WGS detected 3 (7.9%) RIF-resistant genotypes that were missed by Xpert MTB/RIF assay. These strains carried the *rpoB* mutation Val170Phe, which is not targeted by Xpert MTB/RIF, and were found within the same lineage (ie, Delhi-CAS) and transmission chain from participants from Siltie and Mekelle zones, suggesting that Xpert MTB/RIF testing alone may miss detection of clonal strains harboring particular *rpoB* mutations, potentially resulting in inappropriate treatment. In this study, we also detected RIF resistance using WGS that was misclassified as RIF susceptible with pDST in 2 patients. These strains harbored mutations associated with lower level of RIF resistance (ie, Leu430Pro, His445Gly). Similar findings have been reported elsewhere [26, 27]. In 2021, the WHO lowered the critical concentration of RIF from 1.0 µg/mL to 0.05 µg/mL for pDST to detect phenotypes easily missed using previous breakpoints [28].

Twenty-five strains belonging to the Euro-American 4.6.2 lineage harbored the phylogenetic marker for a common MTB sublineage in the Horn of Africa, which also confers capreomycin resistance (*tlyA* Asn236Lys mutation) [20].

MTB lineages 4, followed by lineage 3, was the most common lineage in this study, consistent with previous evidence in Ethiopia [29–31]. At the subnational level, similar frequencies were observed in Tigray [32], and sublineage 4.2 was predominant in a review study [33]. Similar distribution was reported in a study conducted on isolates collected during the previous DRS study, with dominant lineage 4 distribution and lineage 3 equally or more represented than lineage 4 in Gambella and Tigray [30], that is, regions bordering with Sudan and Eritrea, respectively, where lineage 3 is frequent [20, 29]. This is the largest study performing WGS-based transmission analysis in Ethiopia and showed low clustering rates compared to the rest of the African region [22] and also to previous data from the country generated through the MIRU-VNTR approach [33]. These data are not surprising, as WGS has higher resolution power for cluster analysis over conventional typing techniques. Clustering was slightly higher in Somali region than rest of the country, in line with previous studies showing a higher proportion in eastern Ethiopia [33].

There are some limitations to our survey. First, due to budget constraints, WGS was not done for all RIF-susceptible participants. Second, pDST was not performed for all participants due to difficulty in obtaining pure or viable MTBC isolates. However, the use of composite DST including Xpert MTB/RIF assay, LPA, pDST, and WGS enabled us to detect resistance, minimizing the risk that DR-TB was missed or misinterpreted. Third, this survey only included patients diagnosed in health facilities supervised by the National TB Control Program as limited number of private facilities are involved in the clinical management of TB. Despite these limitations, our finding provides a comprehensive report from a nationally representative sample being generalizable to the population.

## CONCLUSIONS

The prevalence of RR-TB among newly diagnosed cases in Ethiopia has remained stable at a low level. The proportion of Hr-TB was higher than RR-TB. Resistance to FLQs and to any other new second-line anti-TB drug was very low in this study, possibly indicating the potential positive impact of the implementation of new BPaL/BPaLM regimens for the treatment of RR/MDR-TB. Further DRS is needed to better understand the magnitude and pattern of resistance to these new regimens. Introducing sequencing technology has a great potential to enhance diagnosis of DR-TB cases including for genotyping, strain typing, and epidemiologic surveillance.

## Supplementary Material

ofaf367_Supplementary_Data

## Supplementary Data

Supplementary materials are available at *Open Forum Infectious Diseases* online. Consisting of data provided by the authors to benefit the reader, the posted materials are not copyedited and are the sole responsibility of the authors, so questions or comments should be addressed to the corresponding author.

## Notes

***Author contributions***. S. M.: Coordination of the study, critical reviewing of the proposal, performing pDST, training, study site monitoring and supportive supervision, compiling geospatial data of study site, cleaning of laboratory and clinical data, data analysis, drafting of the manuscript, compiling feedback, manuscript finalization and approval. M. Geta.: Developing the proposal, member of drug resistance survey technical working group (DRS TWG), training healthcare providers on the data collection, study site monitoring and support, coordinate and monitor the study activities, pDST, coordinate and actively participate in cleaning of laboratory data, quality control testing, analysis, drafting of the manuscript, reviewing and approval of the manuscript. Z. M.: Developing the proposal, member of DRS TWG, coordinate and monitor the study activities, training healthcare providers on the data collection, study site coordination and monitoring, coordinate and actively participate in cleaning of laboratory and clinical data during sample collection, analysis, drafting of the manuscript, reviewing and approval of the manuscript. A. Al.: Study site monitoring and support, culture processing, quality control testing, data cleaning and analysis, reviewing and approval of the manuscript. G. D. and B. Y.: Reviewing of the proposal, study site monitoring and support, pDST, quality control testing, data cleaning and analysis, reviewing and approval of the manuscript. D. F.: Study site monitoring and support, training healthcare providers on the data collection, coordinate and actively participate in cleaning of laboratory and clinical data, analysis, overall data management, reviewing and approval of the manuscript. Y. A.: Reviewing of the proposal, culture processing, study site monitoring and support, data cleaning and analysis, reviewing and approval of the manuscript. M. A.: Reviewing of the proposal, study site monitoring and support, culture processing and line probe assay, quality control testing, data cleaning and analysis, reviewing and approval of the manuscript. E. T.: Drafting of the proposal, study site monitoring and support, training healthcare providers on the data collection, coordinate the study site monitoring and support, pDST, data cleaning and analysis, reviewing and approval of the manuscript. A. K.: Developing the proposal, member of DRS TWG, study site monitoring and support, training healthcare providers on the data collection, pDST, advise the cleaning of laboratory data, analysis, reviewing and approval of the manuscript. Z. Y.: Coordinating the proposal development, initiation of the study, reviewing and approval of the manuscript. A. M.: Study site monitoring and support, pDST, data cleaning, reviewing and approval of the manuscript. H. M.: Study site monitoring and support, pDST, quality control testing, data cleaning, reviewing and approval of the manuscript. B. D.: Study site monitoring and support, culture processing, data cleaning, reviewing and approval of the manuscript. M. T. and W. S.: Drafting of the proposal, study site monitoring and support, culture processing, data cleaning, reviewing and approval of the manuscript. G. S.: Study site monitoring and support, culture processing, data cleaning, reviewing and approval of the manuscript. B. Z. and M. G.: Culture processing, data cleaning, reviewing and approval of the manuscript. G. Ta., S. A., and G. To.: Study site monitoring and support, data interpretation, reviewing and approval of the manuscript. A. A., A. Y. H., Y. M., F. M., and D. A.: Developing the proposal, member of DRS TWG, study site monitoring and support, reviewing and approval of the manuscript. B. F. and T. G.: Providing technical assistance for the protocol development, member of DRS TWG, coordinating and monitoring the study activities, training healthcare providers on the data collection, and reviewing and contributing to the draft manuscript. F. D. M.: Reviewing the proposal, study site monitoring and support, genomic data analysis and interpretation, critical reviewing and approval of the manuscript. D. M. C. and A. D.: Technical assistance for the protocol development, advise the cleaning of laboratory data, data interpretation, critical reviewing and approval of the manuscript. A. M. C.: Technical assistance for the protocol development, advise the cleaning of laboratory data, genomic data analysis and interpretation, critical review and approval of the manuscript. E. K.: Developing the proposal, member of DRS TWG, study site monitoring and support, supervising healthcare providers on the data collection, advise and follow the cleaning of laboratory data, follow and actively participate in the analysis, critical reviewing and approval of the manuscript.

***Acknowledgments.*** The authors would like to thank Ethiopian regional states and regional TB culture laboratories. In particular, we thank the study participants and survey data collectors for their contribution. The WHO Country Office for Ethiopia, and particularly Dr Ismael Hassen, are thanked for their support in facilitating survey implementation, monitoring, and evaluation. We gratefully acknowledge the financial and technical support from the United States Agency for International Development and the Global Fund.

***Financial support.*** This survey was supported in part by the US President’s Emergency Plan for AIDS Relief (PEPFAR) through the Centers for Disease Control and Prevention (cooperative agreement number GH001178). Whole genome sequencing was financially supported by the World Health Organization.
