# Supplementary material for: The Ethiopian Third National Tuberculosis Drug Resistance Survey Incorporating Whole Genome Sequencing
Source: Open Forum Infect Dis. 2025 Jul 21;12(7):ofaf367. doi: 10.1093/ofid/ofaf367 (PMC12278268; doi:10.1093/ofid/ofaf367)
Supplement: ofaf367_Supplementary_Data [file ofaf367_supplementary_data.docx]

**Supplementary** **Table 1**. Socio-behavioral profiles of the study participants

| **Variables** | | **Newly diagnosed** | **Previously treated** | **Total** | ***P-*value** |
| --- | --- | --- | --- | --- | --- |
|  |  | Number (%) | Number (%) | Number (%) |  |
| Khat Chewing | Yes | 378 (18.5) | 55 (23.5) | 433 (19.0) | 0.0654 |
|  | No | 1664 (81.5) | 179 (76.5) | 1843 (81.0) |  |
| Current Smoker | Yes | 144 (7.0) | 22 (9.4) | 166 (7.3) | 0.1905 |
|  | No | 1898 (93.0) | 212 (90.6) | 2125 (92.7) |  |
| Ex-smoker | Yes | 153 (7.5) | 26 (11.1) | 179 (7.9) | 0.0515 |
|  | No | 1889 (92.5) | 208 (88.9) | 2097 (92.1) |  |
| Prisoner/prisoner staff | Yes | 8 (0.4) | 4 (1.7) | 12 (0.5) | 0.0277 |
|  | No | 2034 (99.6) | 230 (98.3) | 2264 (99.5) |  |
| History of incarceration | Yes | 61 (3.0) | 11 (4.7) | 72 (3.2) | 0.1561 |
|  | No | 1981 (97.0) | 223 (95.3) | 2204 (96.8) |  |
| Migrant | Yes | 30 (1.5) | 6 (2.6) | 36 (1.6) | 0.2036 |
|  | No | 2012 (98.5) | 228 (97.4) | 2240 (98.4) |  |
| Education | No formal education | 819 (39.9) | 97 (41.1) | 916 (40.0) | 0.426 |
|  | Primary and secondary | 1084 (52.7) | 127 (53.8) | 1211 (52.9) |  |
|  | Tertiary | 152 (7.4) | 12 (5.1) | 164 (7.2) |  |
| Occupation | Farmer | 633 (30.8) | 78 (33.1) | 711 (31.0) | <0.001 |
|  | Daily Labor | 228 (11.1) | 42 (17.8) | 270 (11.8) |  |
|  | House wife | 362 (17.6) | 43 (18.2) | 405 (17.7) |  |
|  | Student | 399 (19.4) | 19 (8.1) | 418 (18.2) |  |
|  | Merchant | 71 (3.5) | 12 (5.1) | 83 (3.6) |  |
|  | Other | 362 (17.6) | 42 (17.8) | 404 (17.6) |  |
| Number of house hold member | Less than 3 Member | 514 (25.0) | 41 (17.4) | 555 (24.2) | 0.007 |
|  | 3-5 Member | 596 (29.0) | 88 (37.3) | 684 (29.9) |  |
|  | >5 Member | 945 (46.0) | 107 (45.3) | 1052 (45.9) |  |
| No. of Rooms of the main house | Only one Room | 843 (41.0) | 84 (35.6) | 927 (40.5) | 0.116 |
|  | 2-3 Rooms | 975 (47.4) | 126 (53.4) | 1101 (48.1) |  |
|  | >3 Rooms | 222 (10.8) | 22 (9.3) | 244 (10.7) |  |
|  | Not reported | 15 (0.7) | 4 (1.7) | 19 (0.8) |  |
| Main means of transportation | Private car | 20 (1.0) | 1 (0.4) | 21 (0.9) | 0.732 |
|  | Taxi | 149 (7.3) | 13 (5.5) | 162 (7.1) |  |
|  | City bus | 45 (2.2) | 6 (2.5) | 51 (2.2) |  |
|  | Bajaj | 276 (13.4) | 37 (15.7) | 313 (13.7) |  |
|  | Animals | 31 (1.5) | 5 (2.1) | 36 (1.6) |  |
|  | Animal drawn cart | 40 (1.9) | 2 (0.8) | 42 (1.8) |  |
|  | On foot | 1477 (71.9) | 170 (72.0) | 1647 (71.9) |  |
|  | Motorcycle | 17 (0.8) | 2 (0.8) | 19 (0.8) |  |

**Supplementary** **Table 2.** MDR-TB and RR-TB Prevalence estimates obtained during analysis for the different models without imputation but using weighing and accounting for cluster design.

|  | **MDR-TB** | | **RR-TB** | |
| --- | --- | --- | --- | --- |
|  | New | Retreatment | New | Retreatment |
| Cluster-level | 1.01 (0.48 - 1.54) | 6.25 (2.07 - 10.42) | 1.06 (0.53 - 1.60) | 7.1 (2.1 - 12.1) |
| Individual level – RS – no weights | 1.22 (0.76– 1.86) | 8.52(4.85 – 13.6) | 1.08 (0.68 -1.63) | 6.93 (4.01 – 11.00) |
| Individual level – RS – weights | 1.19 (0.71 – 2.01) |  | 1.06 (0.64 – 1.75) |  |
| LR: no weights, no clustering | 1.22(0.70 - 1.74) | 8.52 (4.40 – 12.6) | 1.08 (0.63 – 15.30) | 6.93 (3.65 - 10.20) |
| LR: weights, no clustering | 1.19 (0.64 - 1.75) |  | 1.06 (0.58 – 1.54) |  |
| Robust standard errors no weights | 1.22 (0.65 – 1.79) | 8.52 (4.70 – 12.3) | 1.08 (0.59 – 1.57) | 6.93 (3.54 – 10.32) |
| Robust standard errors and weights | 1.19 (0.59 - 1.81) |  | 1.06 (0.54 – 1.59) |  |
| Imputation model | 1.28 (0.78 - 2.10) | 8.4 (4.68 – 14.65) | 1.07 (0.65 – 1.74) | 6.89 (4.02 – 11.57) |

**Supplementary Table 3:** Rifampicin Resistance Discordance among WGS-tested clinical isolates^*^

| Test method | Total | Xpert MTB/RIF assay  RIF resistance result (n=2248) | | | | Phenotypic RIF DST (n=1552) | | |
| --- | --- | --- | --- | --- | --- | --- | --- | --- |
|  |  | R | S | I | missing | R | S | missing |
| WGS-RIF resistant | 33 | 30 | 3 | 0 | - | 27 | 2 | 4 |
| WGS: RIF-R, INH-R | 29 | - | - | - | - | 24 | 2 | 3 |
| WGS: RIF-R, INH-S | 4 | - | - | - | - | 3 | 0 | 1 |
| WGS: RIF-S | 621 | 1 | 619 | 1 | - | 0 | 611 | 10 |
| Total WGS test | 654 | 31 | 622 | 1 | - | 27 | 613 | 14 |
| WGS missing | 1613 | 5 | 1587 | 2 | 19 | 1 | 911 | 701 |

* WGS was used as tiebreaker for RIF resistance interpretation if there is discordance. WGS=whole genome sequencing, RIF=rifampicin, INH=isoniazid, DST=drug susceptibility testing, R=resistant, S=sensitive, I=indeterminate

**Supplementary** **Table 4**. INH resistance prevalence estimates obtained during analysis for the different models without imputation but using weighing and accounting for cluster design

|  | **INH resistance (any)** | | **INH monoresistant** | |
| --- | --- | --- | --- | --- |
|  | New | **Retreatment** | New | **Retreatment** |
| Cluster-level | 4.99 (6.02 – 17.96) | 11.99 (6.02 – 1796) | 3.76 (2.58 - 4.93) | 4.29 (2.39 – 8.43) |
| Individual level – RS – no weights | 5.33 (4.33 - 6.51) | 13.07 (0.85 - 1896) | 4.12 (3.23 – 5.17) | 4.55 (1.98 – 8.76) |
| Individual level – RS – weights | 4.99 (3.75 - 6.61) |  | 3. 76 (2.75 – 5.13) |  |
| LR: no weights, no clustering | 5.33 (4.28 – 6.40) | 13.07 (0.81 -18.05) | 4.12 (3.18 – 5.05) | 4.55 (1.47 – 7.62) |
| LR: weights, no clustering | 4.99 (3.75 – 6.61) |  | 3.76 (2.83- 4.69) |  |
| Robust standard errors no weights | 5.34 (3.93 – 6.75) | 13.07 (0.84 –17.74) | 4.12 (2.86 -5.38) | 4.55 (1.41 – 7.68) |
| Robust standard errors and weights | 4.99 (3.60 – 6.38) |  | 3.76 (2.61 – 4.91) |  |
| With imputation | 5.35 (4.14-6.880 | 12,5 (8.10-18.88) | 4.15 (3.11 - 5,.53) | 4.41 (1.97 -9.57) |

**Supplementary Table 5:** Isoniazid Resistance Discordance among WGS-tested clinical isolates^*^

| Test method | Total | WGS INH DST (n=654) | | |
| --- | --- | --- | --- | --- |
|  |  | Resistant | Sensitive | missing |
| pDST INH-R | 90 | 72 | 12 | 6 |
| pDST INH-R, RIF-S (Hr-TB) | 63 | 48 | 10 | 5 |
| pDST INH-R, RIF-R | 27 | 24 | 2 | 1 |
| pDST INH-S | 1462 | 2 | 554 | 906 |
| Total pDST test | 1552 | 74 | 566 | 912 |

* phenotypic DST were used as a reference for interpretation of discordant INH resistance. pDST= phenotypic drug susceptibility testing, WGS=whole genome sequencing, INH=isoniazid, RIF=rifampicin, R=resistant, S=sensitive
